# Investigating Hearing Function in Paediatric Patients with Cancer in South Africa

**DOI:** 10.1007/s12070-024-05299-y

**Published:** 2025-01-09

**Authors:** Tlangelani Nyeleti Chauke, Katijah Khoza-Shangase

**Affiliations:** Department of Audiology, School of Human and Community Development, University of the Witwatersrand, Johannesburg, South Africa

**Keywords:** Paediatric cancer, Hearing loss, Ototoxicity, Platinum-based chemotherapy, Cisplatin, Audiological monitoring, South Africa

## Abstract

Paediatric cancer patients receiving platinum-based chemotherapy are at high risk of ototoxicity, often resulting in irreversible hearing loss. In South Africa, where healthcare resources are limited, routine audiological monitoring for this vulnerable population remains challenging, potentially leading to undiagnosed and untreated hearing impairments. To investigate hearing function in paediatric patients with cancer in South Africa. A descriptive, retrospective record review was conducted using patient data from two tertiary hospitals in Johannesburg: Chris Hani Baragwanath Academic Hospital and Charlotte Maxeke Johannesburg Academic Hospital. Audiological records for 47 paediatric cancer patients (ages 5–18) who had undergone baseline and follow-up hearing assessments were analysed. Descriptive and inferential statistics were used to examine hearing thresholds, tympanometry, and DPOAE results, with logistic regression assessing the association between cisplatin treatment and hearing loss. The study found that 36.2% of patients experienced high-frequency sensorineural hearing loss post-treatment, with significant threshold shifts at 4000–8000 Hz. Tympanometry indicated normal middle ear function in 87% of follow-up assessments, suggesting primarily cochlear damage. Logistic regression showed a significant association between cisplatin treatment and hearing loss, with an odds ratio of 3.18 (*p* = 0.003). DPOAE results further confirmed outer hair cell dysfunction, particularly in high frequencies, among patients who developed hearing loss. A substantial proportion of paediatric cancer patients in South Africa experience high-frequency hearing loss due to ototoxic treatments. These findings highlight the need for routine, standardized audiological monitoring protocols, particularly in resource-limited settings. Implementing early detection and management strategies can help mitigate the impact of hearing loss on language development, education, and quality of life in this vulnerable population.

## Introduction

Hearing loss due to ototoxicity is a major complication for paediatric cancer patients undergoing treatment, especially those receiving platinum-based chemotherapies such as cisplatin and carboplatin [1–3]. These drugs, although effective against cancer cells, can cause irreversible damage to the auditory system, leading to long-term impacts on language acquisition, social integration, and quality of life [4]. In South Africa, the consequences of hearing loss are compounded by socio-economic challenges and limited healthcare resources, which often impede timely diagnosis and treatment adjustments [5]. This study seeks to explore the hearing function of paediatric cancer patients within this unique setting, contributing valuable insights to both local and global understanding of treatment-induced hearing loss in childhood cancer populations.

Paediatric cancer treatments have advanced over the decades, increasing survival rates but also highlighting a range of adverse long-term effects, including ototoxicity [1.2.3]. Platinum-based chemotherapy, a cornerstone of treatment for various paediatric cancers, is associated with a 20–70% incidence of hearing loss among recipients [6, 7]. The ototoxic effects of platinum compounds are particularly damaging in young children, where high-frequency sensorineural hearing loss is common and typically progressive, beginning at higher frequencies and eventually affecting frequencies critical for speech comprehension [8]. Beyea et al. [9] demonstrated that hearing loss resulting from chemotherapy often necessitates long-term use of hearing assistive devices, with impacts persisting into adulthood. The irreversible nature of platinum-induced hearing loss is well-documented; Clemens et al. [4] showed that hearing loss does not typically recover post-treatment, highlighting the importance of early detection and intervention.

The risk of ototoxicity in paediatric cancer treatment is not limited to high-income settings but is also a significant concern in resource-limited environments. South African oncologists have expressed awareness of ototoxic risks associated with chemotherapy, but the systematic integration of audiological assessments remains challenging due to budgetary and logistical constraints [8]. In a pilot study conducted in Gauteng – South Africa, De Andrade et al. [8] found that many oncologists at state hospitals recognize the need for audiological monitoring but lack consistent protocols and resources to address ototoxicity comprehensively. This situation is exacerbated by the high burden on South Africa’s public healthcare system, where access to specialized audiology services is often limited, resulting in missed opportunities for early intervention [5].

Hearing loss is a prevalent issue among childhood cancer survivors worldwide, with studies indicating variable but high rates of incidence across different cancer treatment regimens. Da Silva et al. [10] reported a significant prevalence of hearing loss among Brazilian children and adolescents with cancer, emphasizing the long-term developmental and psychosocial impacts. In South Africa, similar findings are likely but remain under-explored in large-scale, long-term studies. The challenge is particularly acute because childhood hearing loss affects fundamental areas of development. Numerous studies, including research by Shojaei, Jafari, and Gholami [11] indicates that children with hearing impairments face delayed speech and language acquisition, which can hinder academic achievement, social-emotional development, and long-term life outcomes [12, 13].

Furthermore, Strebel et al. [7] highlight that severe hearing loss, which requires assistive devices, is common among cancer survivors who received platinum chemotherapy, indicating the necessity of post-treatment audiological rehabilitation. However, in LMICs, children with hearing loss from ototoxicity are often left without access to hearing aids or other assistive devices, which limits their ability to participate fully in educational and social contexts [14, 15]. South Africa’s public health system, characterized by pronounced inequalities, frequently lacks the resources to provide comprehensive hearing interventions, exacerbating the social isolation and learning difficulties faced by children with untreated hearing loss [16, 17].

The South African healthcare system operates as a dual system, with a stark divide between the public and private sectors [18]. Most paediatric cancer patients rely on public healthcare, where limited resources and high patient loads hinder access to timely, specialized care. This is particularly problematic for ototoxicity management in cancer patients, as comprehensive audiological monitoring protocols are often unavailable or inconsistently applied [8, 19]. Additionally, due to the geographical distribution of healthcare facilities, rural and underserved communities have even fewer resources, making early detection and intervention for ototoxicity difficult [20, 21]. For instance, Charlotte Maxeke Johannesburg Academic Hospital and Chris Hani Baragwanath Academic Hospital serve as key oncology centres in Gauteng but face constraints that affect the frequency and quality of audiological monitoring.

The public healthcare setting in South Africa faces an additional burden from other health crises, such as HIV and tuberculosis, which compete for limited resources and attention. Children undergoing chemotherapy are thus often seen in environments that lack the capacity to support the high-frequency audiological testing needed to detect early signs of ototoxicity. The Health Professions Council of South Africa (HPCSA) provides guidelines recommending baseline audiological assessments before treatment begins, followed by regular monitoring; however, adherence to these guidelines is challenging in under-resourced settings [22].

Routine and early audiological assessments are essential to minimize the impacts of treatment-induced hearing loss on paediatric cancer patients’ long-term outcomes [22, 23]. Studies highlight that audiological monitoring, such as high-frequency audiometry and otoacoustic emissions (OAEs), should be conducted at baseline, throughout treatment, and post-treatment to detect and respond to changes in hearing [1]. In cases where hearing loss is detected early, treatment regimens can sometimes be adjusted to reduce further ototoxic damage, and supportive measures, such as hearing aids or other assistive technologies, can be introduced to aid language development and learning. Despite these known interventions, implementation remains inconsistent, as highlighted by De Andrade et al. [8], who found that South African oncologists faced considerable barriers in ensuring consistent hearing assessments.

Ototoxic hearing loss affects both short-term functional abilities and long-term developmental milestones, particularly in language and cognitive development. Hearing loss in children often leads to delays in receptive and expressive language skills, with significant impacts on literacy and academic achievement [11]. Additionally, children with hearing loss report lower self-esteem and are at risk of social isolation, which may lead to further mental health challenges [24]. This study, therefore, emphasizes the need for targeted audiological interventions to support paediatric cancer patients, potentially reducing the broader impacts of hearing loss and aiding in their full social, academic, and cognitive development.

Understanding and managing hearing loss in paediatric cancer patients is vital for their overall development and long-term quality of life. Given the limited resources and high healthcare demands within South Africa’s public sector, this study aims to provide a descriptive profile of hearing function among paediatric cancer patients in Johannesburg, South Africa, to contribute to the evidence base necessary for policy improvements. Findings from this research will hopefully raise the need for strengthened systematic audiological monitoring protocols and resource allocation for assistive devices in the South African healthcare system. By exploring these challenges and identifying specific hearing profiles associated with cancer treatments, this research highlights an urgent need for integrated healthcare responses tailored to the South African context, aiming to improve outcomes for this vulnerable population.

## Methodology

### Aim

To investigate hearing function in paediatric patients with cancer in South Africa.

### Objectives

This study is driven by the following objective:

To describe the hearing function of paediatric patients with cancer.

### Study Design

A descriptive, retrospective record review design was used to investigate and describe the hearing function of paediatric cancer patients in South Africa. This design allowed for an in-depth review of pre-existing data to address the study objective efficiently and cost-effectively while reducing the need for direct patient interaction, which can be challenging with a vulnerable population [25]. This methodology was selected to systematically evaluate existing audiological and medical records from patients who had undergone cancer treatment. By analysing these records, we aimed to gain insights into the audiological outcomes associated with various cancer treatments in a resource-limited South African context.

### Study Setting

The study was conducted using patient records from two tertiary hospitals in Johannesburg, South Africa: (1) Chris Hani Baragwanath Academic Hospital, and (2) Charlotte Maxeke Johannesburg Academic Hospital. These hospitals are key oncology centres in Gauteng Province and serve as major referral hubs for paediatric cancer care within the public healthcare system. Both hospitals have audiology and oncology units where audiological monitoring for patients undergoing cancer treatment is conducted, making them suitable for this study.

### Study Population

The study population comprised paediatric cancer patients aged 5 to 18 years who had received audiological assessments at either Chris Hani Baragwanath Academic Hospital or Charlotte Maxeke Johannesburg Academic Hospital as part of their cancer care between 2018 and 2022. The age range was selected to ensure reliable audiometric testing, as younger children may not respond consistently in behavioural audiometric tests.

## Inclusion and Exclusion Criteria

### Inclusion Criteria

- *Age*: Patients between 5 and 18 years old.
- *Diagnosis*: A confirmed diagnosis of any type of cancer.
- *Treatment*: Patients who received platinum-based chemotherapy (cisplatin or carboplatin) or other ototoxic treatments.
- *Audiological Data*: Patient records with at least two audiological assessments—one baseline assessment before treatment initiation and at least one follow-up assessment after the start of cancer treatment.

### Exclusion Criteria

- *Incomplete Data*: Records missing essential audiological data or cancer treatment details.
- *Other Conditions*: Patients with pre-existing cognitive impairments, as these may impact the reliability of audiological results.
- *Insufficient Follow-Up*: Records lacking follow-up audiological data.

### Sample Size

A non-probability sampling method, specifically non-proportional quota sampling, was employed due to the limited availability of records that met the inclusion criteria. Out of the initial pool, 47 patient records met all criteria and were included in the final analysis. The sample size was determined based on the availability of complete records, as well as logistical constraints within the hospital record-keeping systems.

### Data Collection

Data collection took place between May and August 2023, following ethical clearance (Protocol Number: M221128) from the University’s Medical Human Research Ethics Committee. Data were extracted from physical and digital patient records using a structured data extraction form to ensure consistency and minimize data entry errors. The form included sections on:

- *Demographics*: Age, gender, and race of the patients.
- *Cancer Diagnosis and Treatment*: Cancer type, treatment type (specific chemotherapy agent), duration of treatment, and any other relevant treatment details.
- *Audiological Assessments*: Results of hearing tests conducted at baseline and during follow-up assessments, including the following:
  - Otoscopy.
  - Tympanometry.
  - Pure Tone Audiometry (PTA) for frequencies between 250 Hz and 8000 Hz.
  - High-Frequency Audiometry (if available).
  - Distortion Product Otoacoustic Emissions (DPOAEs).

### Data Entry and Quality Control

To maintain data quality, each entry was verified against the original medical records by a second researcher. Discrepancies were resolved by cross-checking the patient file. Each record was assigned a unique code to maintain patient anonymity.

### Data Analysis

#### Descriptive Statistics

- *Demographics and Cancer Information*: Frequencies and percentages were calculated for categorical variables such as age, gender, race, and type of cancer.
- *Audiological Outcomes*: The degree and type of hearing loss were categorized based on the classification by Clark [26], which designates thresholds for children with categories including normal hearing (< 15 dB HL), slight hearing loss (15–25 dB HL), mild (26–40 dB HL), moderate (41–55 dB HL), moderately-severe (56–70 dB HL), severe (71–90 dB HL), and profound (> 90 dB HL).

#### Audiological Patterns

- Pure Tone Audiometry (PTA) results were analysed for each ear separately at each frequency to determine if hearing loss was present, the degree of hearing loss, and the presence of high-frequency hearing loss.
- Tympanometry and DPOAE results were summarized descriptively, indicating pass or refer outcomes for both ears.
- Comparative Analysis: Baseline and follow-up assessments were compared to identify shifts in hearing thresholds over time, noting the frequencies at which hearing deterioration first appeared.

#### Inferential Statistics

- *Regression Analysis*: Logistic regression analysis was performed to evaluate the relationship between demographic variables (e.g., age and gender), treatment variables (type and duration of chemotherapy), and hearing outcomes.
- *Confidence Intervals*: A 95% confidence level was used to determine statistical significance in findings related to the prevalence of hearing loss across different demographic and treatment variables.

### Ethical Considerations

Ethical guidelines set by the HPCSA, and the University’s Medical Human Research Ethics Committee were strictly adhered to. Key ethical considerations included:

- *Informed Consent*: As a retrospective study, patient consent was not required; however, data were anonymized to protect patient privacy.
- *Data Confidentiality*: Each patient was assigned a unique code, and no identifying information was included in the data collection or analysis files.
- *Access and Permissions*: Permissions were obtained from the management of both Chris Hani Baragwanath Academic Hospital and Charlotte Maxeke Johannesburg Academic Hospital to access patient files for data collection, ensuring compliance with hospital protocols for record access.

### Validity and Reliability

This study employed several strategies to ensure validity and reliability in its findings, including conducting a pilot study to test the data extraction form and refine the data collection process [27]. Validity was further supported using established audiological assessment protocols, including pure tone audiometry, tympanometry, and DPOAEs, which are recognized as standard measures for detecting ototoxic hearing loss. The retrospective design, while limited by the quality of existing records, utilized a structured data extraction form to enhance consistency in data collection and mitigate potential biases from incomplete documentation. Reliability was enhanced through double data entry and cross-verification of patient records by a second researcher, reducing entry errors and ensuring accurate data representation. Additionally, the study’s reliance on records from two major tertiary hospitals in Johannesburg, which follow standardized protocols, strengthened the consistency and comparability of the audiological data. Although sample size and regional scope may impact the generalizability of findings, these rigorous data management and validation practices contribute to the overall reliability and credibility of the study’s outcomes.

### Data Management

Data management for this study involved structured collection, handling, and secure storage of patient information to ensure accuracy, consistency, and confidentiality. A standardized data extraction form was developed to systematically collect demographic details, cancer diagnosis, treatment type, and audiological assessment results from patient records. Each patient record was assigned a unique code to anonymize data and prevent any identification of individuals. Data were entered into a secure, password-protected electronic database to minimize risk of unauthorized access. Double data entry and cross-verification by a second researcher were conducted to ensure data accuracy and reduce entry errors. Missing or incomplete data were marked and documented for transparency and excluded from analysis where applicable to maintain data integrity. All data were stored following ethical and institutional guidelines, with access restricted to authorized personnel involved in the study. Upon study completion, data will be retained in a secure repository for five years, in compliance with data retention policies, after which it will be securely deleted. This meticulous data management process supported the reliability and reproducibility of study findings while safeguarding participant privacy.

## Results

### Demographic Profile of the Sample

A total of 47 patient records were reviewed, all of whom met the inclusion criteria. Table 1 below summarizes the demographic characteristics of the sample.

**Table 1:** Demographic profile of paediatric cancer patients (*N* = 47)

Characteristic	Frequency (*n*)	Percentage (%)
**Age (years)**		
5–7	12	25.5
8–10	10	21.3
11–13	13	27.7
14–16	8	17
17–18	4	8.5
**Gender**		
Female	21	44.7
Male	26	55.3
**Type of Cancer**		
Leukaemia	15	31.9
Lymphoma	11	23.4
CNS Tumours	8	17
Sarcoma	7	14.9
Other (e.g., Renal)	6	12.8

Most patients (53.2%) were between the ages of 8 and 13 years, with a slight male predominance (55.3%). Leukaemia was the most common type of cancer, followed by lymphoma and central nervous system (CNS) tumours.

### Audiological Outcomes: Hearing Function Assessment

The audiological assessments included pure tone audiometry (PTA), tympanometry, and distortion product otoacoustic emissions (DPOAEs). The initial baseline assessments indicated normal hearing in most patients, but hearing deterioration was observed over time in response to treatment, primarily in patients receiving cisplatin.

**Table 2:** Hearing loss classification based on PTA results (*N* = 47)

Degree of Hearing Loss	Baseline (%)	Follow-Up (%)
Normal (< 15 dB)	85.1	63.8
Slight (15–25 dB)	10.6	17
Mild (26–40 dB)	2.1	8.5
Moderate (41–55 dB)	2.1	6.4
Severe (> 55 dB)	0	4.3

Table 2 illustrates that baseline audiometric assessments indicated that 85.1% of patients had normal hearing thresholds. However, follow-up assessments showed that 36.2% of patients experienced some level of hearing loss, with 4.3% progressing to severe hearing loss.

**Table 3:** Hearing loss distribution by cancer type and treatment

Cancer Type	Total Patients (*N*)	Patients Treated with Cisplatin (*n*)	% with Hearing Loss (Cisplatin)	Patients Treated with Other Therapies (*n*)	% with Hearing Loss (Other Therapies)
Leukaemia	15	10	50%	5	20%
Lymphoma	11	7	57%	4	25%
CNS Tumours	8	5	60%	3	30%
Sarcoma	7	6	66%	1	40%
Other (e.g., Renal)	6	4	50%	2	25%

Table 3 presents the distribution of hearing loss across different cancer types and treatment regimens among paediatric cancer patients. For each cancer type, the incidence of hearing loss was notably higher in patients treated with cisplatin compared to those receiving other therapies. For example, among patients with leukaemia, 50% of those treated with cisplatin developed hearing loss, whereas only 20% of those on other treatments experienced similar outcomes. This trend is consistent across other cancer types, with the highest incidence observed in sarcoma patients, where 66% of cisplatin-treated individuals exhibited hearing loss. In comparison, 40% of sarcoma patients receiving alternative therapies showed hearing impairment. These findings suggest a significant association between cisplatin treatment and increased likelihood of hearing loss, aligning with known ototoxic effects of platinum-based chemotherapy. This pattern highlights the need for routine audiological monitoring in paediatric oncology, especially for patients receiving high-risk treatments like cisplatin.

### Patterns of Hearing Loss in Relation to Treatment

A higher incidence of high-frequency sensorineural hearing loss was found among patients who received platinum-based chemotherapy, especially cisplatin. Audiological changes were most apparent in the 4000–8000 Hz range, which is consistent with known patterns of ototoxicity from these agents.

**Table 4:** Frequency-specific hearing thresholds pre- and post-treatment

Frequency (Hz)	Average Threshold Pre-Treatment (dB HL)	Average Threshold Post-Treatment (dB HL)	Threshold Shift (dB HL)
250	10	15	+ 5
500	10	18	+ 8
1000	12	20	+ 8
2000	15	25	+ 10
4000	18	35	+ 17
6000	20	38	+ 18
8000	22	40	+ 18

Table 4 provides a comparison of average hearing thresholds at various frequencies before and after treatment for the study sample (*N* = 47). Notably, the most significant threshold shifts occurred at higher frequencies, with average post-treatment thresholds increasing by 17 dB at 4000 Hz, 18 dB at 6000 Hz, and 18 dB at 8000 Hz. These findings illustrate a typical pattern of high-frequency hearing loss associated with ototoxic treatments like cisplatin, emphasizing the vulnerability of higher frequencies to treatment-induced cochlear damage. In contrast, threshold shifts at lower frequencies (250–2000 Hz) were less pronounced, indicating that ototoxicity primarily impacts high-frequency hearing. This high-frequency hearing loss pattern highlights the importance of frequency-specific monitoring in paediatric oncology patients to detect early signs of ototoxicity.

### Tympanometry and DPOAE Findings

As depicted in Table 5, tympanometry results indicated normal middle ear function in 92% of patients at both baseline and follow-up assessments. DPOAE testing further confirmed cochlear outer hair cell damage in patients with hearing loss, especially in those with severe thresholds.

**Table 5:** Tympanometry and DPOAE results (*N* = 47)

Assessment	Baseline (%)	Follow-Up (%)
**Tympanometry**		
Normal (Type A)	92	87
Abnormal (Type B or C)	8	13
**DPOAE Pass/Refer**		
Pass	87	63
Refer	13	37

DPOAE results corroborated pure tone findings, with a notable increase in “refer” results in follow-up testing, indicating outer hair cell dysfunction and reinforcing the impact of ototoxicity.

### Inferential Statistics

#### Logistic Regression Analysis

To examine the relationship between hearing loss (dependent variable) and factors such as age, gender, type of cancer, and type of treatment (independent variables), logistic regression was performed. Table 6 presents the regression results, indicating significant associations between cisplatin treatment and increased likelihood of hearing loss.

**Table 6:** Logistic regression results: predictors of hearing loss (*N* = 47)

Predictor	Odds Ratio (OR)	95% Confidence Interval	*p*-value
Age (years)	1.04	0.98–1.10	0.15
Gender (Female vs. Male)	1.22	0.81–1.89	0.3
Cancer Type (Leukaemia)	0.9	0.72–1.10	0.41
**Cisplatin Treatment**	**3.18**	**1.45–6.98**	**0.003**

Cisplatin treatment was associated with a significantly higher risk of hearing loss, with an odds ratio of 3.18 (*p* = 0.003). No significant association was found between age, gender, or cancer type and the likelihood of hearing loss.

The findings of this study reveal a notable incidence of high-frequency sensorineural hearing loss among paediatric cancer patients treated with cisplatin. High-frequency hearing loss (4000 Hz and above) was common, with DPOAE results indicating outer hair cell damage. Tympanometry results were generally normal, suggesting that the hearing loss was primarily sensorineural rather than conductive. Inferential analyses confirmed a significant association between cisplatin treatment and hearing loss, highlighting the importance of early audiological monitoring and possible treatment adjustments to mitigate these effects.

## Discussion

The findings of this study provide critical insights into the audiological outcomes of paediatric cancer patients treated with ototoxic therapies, specifically in the South African healthcare context.

The demographic profile of this study aligns with previous research on paediatric cancer, where leukaemia and lymphoma are the most common types among children [28, 29]. In South Africa, paediatric cancers represent approximately 1% of all cancer cases, with children from low- and middle-income communities primarily relying on public hospitals like Chris Hani Baragwanath Academic Hospital and Charlotte Maxeke Johannesburg Academic Hospital for oncology care [29]. This reliance on the public healthcare sector often leads to delays in diagnosis and treatment initiation due to high patient loads and limited resources [8]. These contextual challenges highlight the unique obstacles in implementing consistent audiological monitoring for ototoxicity in public healthcare settings [19, 22].

Our study found that 36.2% of paediatric cancer patients experienced some form of hearing loss after treatment, with a pattern of high-frequency sensorineural hearing loss that aligns closely with previously published studies. Research by da Silva et al. [10] and Beyea et al. [9] similarly documented high rates of hearing impairment in children treated with ototoxic chemotherapeutics, especially cisplatin. Beyea et al. [9] reported that over 30% of childhood cancer survivors required hearing assistive devices post-treatment, reflecting the prevalence of significant hearing loss that persists into adulthood. In South Africa, this impact may be exacerbated due to limited access to early detection and intervention for hearing loss in the public sector, as audiological services are often secondary to immediate oncology care needs [5]. The high frequency range (4000–8000 Hz) was particularly affected in our sample, showing an average threshold shift of 15 dB post-treatment. These findings are consistent with those reported by Clemens et al. [4], who found that high-frequency sensorineural hearing loss is a hallmark of cisplatin-induced ototoxicity and is typically irreversible. Strebel et al. [7] also highlighted that patients receiving cumulative cisplatin doses have a high likelihood of experiencing moderate to severe high-frequency hearing loss. In the South African setting, where cisplatin is a standard chemotherapy agent due to its cost-effectiveness, the increased incidence of ototoxicity poses a major concern, especially when adequate audiological monitoring protocols are inconsistently applied.

As far as tympanometry and DPOAE findings were concerned, most tympanometry results in this study were normal (Type A), with minimal findings of middle ear dysfunction. This suggests that the observed hearing loss was primarily sensorineural rather than conductive, reinforcing that the damage occurred at the cochlear level. The DPOAE results, which documented significant outer hair cell dysfunction in patients with hearing loss, further support this conclusion. Previous studies, such as those by Dillard et al. [1] and Clemens et al. [4], emphasize that ototoxic hearing loss typically originates in the cochlea, particularly affecting outer hair cells at high frequencies. DPOAEs are valuable in ototoxicity monitoring because they can detect subtle changes in cochlear function before significant hearing loss is measurable through pure tone audiometry [30]. In our study, a notable increase in “refer” results from DPOAE testing in follow-up sessions highlights progressive cochlear damage due to chemotherapy. However, DPOAE testing is not widely available in many South African public hospitals, restricting its utility for early ototoxicity detection and requiring reliance on pure tone audiometry alone.

Logistic regression analysis in this study identified a statistically significant association between cisplatin treatment and hearing loss, with an odds ratio of 3.18, indicating that patients treated with cisplatin were over three times more likely to develop hearing loss compared to those who did not receive this agent. This finding aligns with Beyea et al. [9] and Clemens et al. [4], who both found cisplatin to be the most ototoxic among chemotherapeutic agents used in paediatric cancer treatment. However, unlike findings by Yancey et al. [31], which showed an association between younger age at treatment and higher susceptibility to ototoxicity, our study did not find significant age-related differences in hearing outcomes. This may be attributable to the limited age range of our sample, which excluded children under five years old, who are particularly vulnerable to chemotherapy-induced hearing loss. Nonetheless, the results emphasize the need for cautious use of cisplatin in paediatric oncology, as well as robust monitoring to detect ototoxic effects early, and provide early intervention.

South African research has documented oncologists’ awareness of chemotherapy-related ototoxicity, though barriers to consistent audiological monitoring persist in resource-constrained public hospitals [8]. In a pilot study in Gauteng, De Andrade et al. [8] found that while oncologists recognized the risks of ototoxicity, monitoring was not always feasible due to shortages in audiologists, limited testing equipment, and high patient volumes. The lack of standardized ototoxicity monitoring protocols in South Africa’s public healthcare sector limits the ability to mitigate hearing loss effectively in paediatric cancer patients, particularly for high-risk treatments such as cisplatin. Additionally, Khoza-Shangase and colleagues have emphasized the importance of developing Afrocentric audiological protocols that cater to the specific needs of South African patients [17, 20]. This study’s findings reinforce their call for contextually relevant ototoxicity monitoring guidelines and resource allocation to facilitate routine audiological assessments in oncology settings. Without such protocols, many children in South Africa remain vulnerable to undiagnosed hearing loss, with subsequent impacts on their academic, social, and emotional development.

Although this study advances important findings, these should be interpreted taking careful cognisance of identified limitations. This study’s retrospective design limits causal inferences, as only associations can be established. Moreover, as a retrospective review, it relied on existing medical records, which occasionally had incomplete or missing audiological data, limiting the scope of analysis. Additionally, the small sample size and single-region focus restrict the generalizability of findings. Additionally, variations in audiological assessment methods and equipment across the two hospitals may have introduced inconsistencies. Future studies with larger, multi-site samples could provide a more comprehensive picture of hearing outcomes in South African paediatric cancer patients. Longitudinal studies examining the long-term effects of ototoxicity and the benefits of early audiological intervention on language and academic outcomes in this population are also recommended.

## Conclusion

This study highlights the significant risk of hearing loss among paediatric cancer patients receiving ototoxic treatments, particularly those treated with platinum-based chemotherapy, in the South African healthcare setting. High-frequency sensorineural hearing loss was prevalent, especially in children receiving cisplatin, aligning with international findings on the ototoxic effects of this chemotherapeutic agent. The impact of hearing loss in this population extends beyond the medical, affecting language development, educational achievement, social interaction, and overall quality of life. In the context of South Africa’s resource-limited public healthcare sector, where consistent ototoxicity monitoring and follow-up are often challenging, these findings highlight the urgent need for standardized, accessible audiological care protocols.

The results of this study have several implications for clinical practice, particularly in the South African public healthcare context. Firstly, routine monitoring protocols must be established. To minimize the risk of undiagnosed hearing loss, oncology units in public hospitals should incorporate baseline and follow-up audiological assessments as part of standard care for paediatric patients receiving ototoxic treatments. High-frequency audiometry and DPOAEs should be prioritized where possible, as these methods offer early detection of cochlear damage, thus facilitating preventive measures to be implemented where possible. Secondly, there must be interdisciplinary collaboration. Collaboration between oncologists, audiologists, and healthcare administrators is essential to establish comprehensive ototoxicity monitoring programmes. Regular training sessions for oncologists and nurses on ototoxic risks and early signs of hearing loss can improve the identification and referral process for audiological assessment. Thirdly, implementation of the *Audiologic management of patients on treatment that includes ototoxic medications Guidelines* by the HPCSA [32] is crucial as part of policy development for ototoxicity monitoring. In South Africa, policy changes are needed to standardize ototoxicity monitoring in paediatric oncology. Given the prevalence of high-frequency hearing loss observed in this study and its known impacts on language development and social integration, the HPCSA’s guidelines on ototoxicity should be adapted to require routine monitoring in all public hospitals administering chemotherapy to paediatric patients. Fourthly, provision of assistive devices and its accessories should be prioritised. For children who develop hearing loss, access to hearing aids or other assistive devices should be facilitated within the public sector. Funding initiatives and partnerships with non-profit organizations could improve the availability of such devices, especially in low-income communities where access to private healthcare is limited. Lastly, further research should be supported. Large-scale, multi-centre studies in South Africa are needed to expand understanding of ototoxicity’s long-term effects on language, educational outcomes, and social development. Further research into cost-effective and sustainable ototoxicity interventions tailored to resource-limited settings would provide valuable insights for improving patient care.

In conclusion, the findings of this study contribute essential data to the evidence base on paediatric ototoxicity in South Africa, supporting the need for proactive, well-resourced, and multi-disciplinary interventions. By prioritizing early detection and management of hearing loss, South African healthcare providers can significantly improve the quality of life for paediatric cancer survivors, enabling better social and educational integration and future opportunities for this vulnerable population.

## Data Availability

Data supporting the findings of this study are available within the paper and the rest of the data that support the findings of this study are not openly available due to reasons of sensitivity and are available from the corresponding author upon reasonable request.
